# Tumefactive Demyelinating Lesion Induced by Infliximab in a Patient With Rheumatoid Arthritis: A Case Report

**DOI:** 10.1002/ccr3.71144

**Published:** 2025-10-09

**Authors:** Shahla Mohammed Saeed Rasul, Zana Abdulrahman Mohammed, Aran Asaad Adiban, Yahya Jawad Kadhm

**Affiliations:** ^1^ Branch of Clinical Sciences, College of Medicine University of Sulaimani Sulaimaniyah Iraq

**Keywords:** demyelination, infliximab, magnetic resonance imaging, rheumatoid arthritis, tumor necrosis factor

## Abstract

Tumor Necrosis Factor‐alpha (TNF‐α) inhibitors have been successfully used to treat rheumatoid arthritis (RA), psoriatic and ankylosing arthritis, and inflammatory bowel disease. While these inhibitors effectively decrease the inflammatory activity of immune‐related disorders, they have been associated with central nervous system (CNS) and peripheral demyelination. In this study, we report a 44‐year‐old female patient with RA who developed neurological symptoms after a course of infliximab therapy due to tumefactive demyelinating lesions (TDLs). Imaging findings led to discontinuation of the drug and treatment with corticosteroids, resulting in clinical improvement. This case underscores the importance of monitoring neurological side effects during TNF‐α therapy and early intervention when symptoms occur. Tumor necrosis factor‐alpha inhibitors, such as infliximab, can unmask or induce demyelinating lesions, even in patients without prior multiple sclerosis. Prompt MRI evaluation and infliximab discontinuation, followed by corticosteroids, can lead to clinical improvement. Neurological assessment is recommended before initiating TNF‐α inhibitors, especially in patients with risk factors for demyelination.

**Summary**

- Tumor necrosis factor‐alpha inhibitors, such as infliximab, can unmask or induce demyelinating lesions, even in patients without prior multiple sclerosis.
- Prompt MRI evaluation and infliximab discontinuation, followed by corticosteroids, can lead to clinical improvement.
- Neurological assessment is recommended before initiating TNF‐α inhibitors, especially in patients with risk factors for demyelination.

## 1 Introduction

Rheumatoid arthritis (RA) is a chronic disease characterized by immune‐mediated inflammatory synovitis, leading to joint cartilage and bone destruction. The inflammatory cells of synovial RA release numerous cytokines, including tumor necrosis factor‐alpha (TNFα), interleukin 1 (IL‐1), and IL‐6 [1].

Tumor necrosis factor (TNF) antagonists, such as infliximab, are widely used in the management of rheumatoid arthritis (RA) due to their potent anti‐inflammatory effects [2]. These biological agents have revolutionized RA treatment by significantly reducing disease activity and improving the quality of life in affected patients. However, TNF antagonists have also been linked to rare but serious neurological side effects, including demyelinating events that affect the central nervous system (CNS) [3]. Although various theories have been proposed, the mechanisms underlying the predisposition to demyelination or the exacerbation of existing demyelination in RA patients treated with TNFα antagonists remain poorly understood [4].

Infliximab, a chimeric monoclonal antibody that precisely and with high affinity binds to soluble and trans‐membrane TNFα and neutralizes the cytokine, is currently approved for treating RA. Then, infliximab at a dose rate of 3.0–10 mg/kg can reduce synovial inflammation, bone resorption, and cartilage degradation. In addition to establishing the safety and efficacy of infliximab, clinical research has also provided insights into the complex cellular and cytokine‐dependent pathways involved in the pathophysiology of RA, including evidence that supports TNFα involvement in cytokine regulation, cell recruitment, angiogenesis, and tissue destruction [5].

## 2 Case History/Examination

A 44‐year‐old female with a nine‐year history of RA presented to Shar Teaching Hospital, Sulaimaniyah, Iraq, with new neurological symptoms following infliximab therapy. Her RA had been managed with methotrexate, sulfasalazine, prednisolone, and hydroxychloroquine for 9 years before switching to infliximab due to inadequate disease control. There was no washout period before switching to infliximab. After receiving nine doses of infliximab over 1 year, the patient's neurological symptoms first appeared 2 months before presentation. Initially, she experienced headache and neck pain, followed by numbness and weakness in her right upper limb.

## 3 Differential Diagnosis, Investigations and Treatment

A nerve conduction study led to the diagnosis of carpal tunnel syndrome, for which she underwent surgery. Post‐operatively, her symptoms persisted and extended to her right lower limb. The brain's magnetic resonance imaging (MRI) revealed a left (LT) fronto‐parietal complex cystic mass, indicating definite CNS involvement. This prompted the discontinuation of infliximab due to the suspicion that the TNF antagonist might be linked to the new neurological findings. Other diagnostic tests were performed to exclude other possible causes, including negative results for toxoplasmosis immunoglobulin M (IgM) and positive IgG antibodies, as well as the Interferon Gamma Release Assay (IGRA) test. A cerebrospinal fluid (CSF) sample was obtained and analyzed for the presence of oligoclonal bands, myelin oligodendrocyte glycoprotein immunoglobulin G (MOG‐IgG), and aquaporin‐4 (AQP4) antibodies. A serum sample was also tested for MOG‐IgG and AQP4 Antibodies. All assays yielded negative results.

## 4 Conclusion and Results (Outcome and Follow‐Up)

**FIGURE 1:**
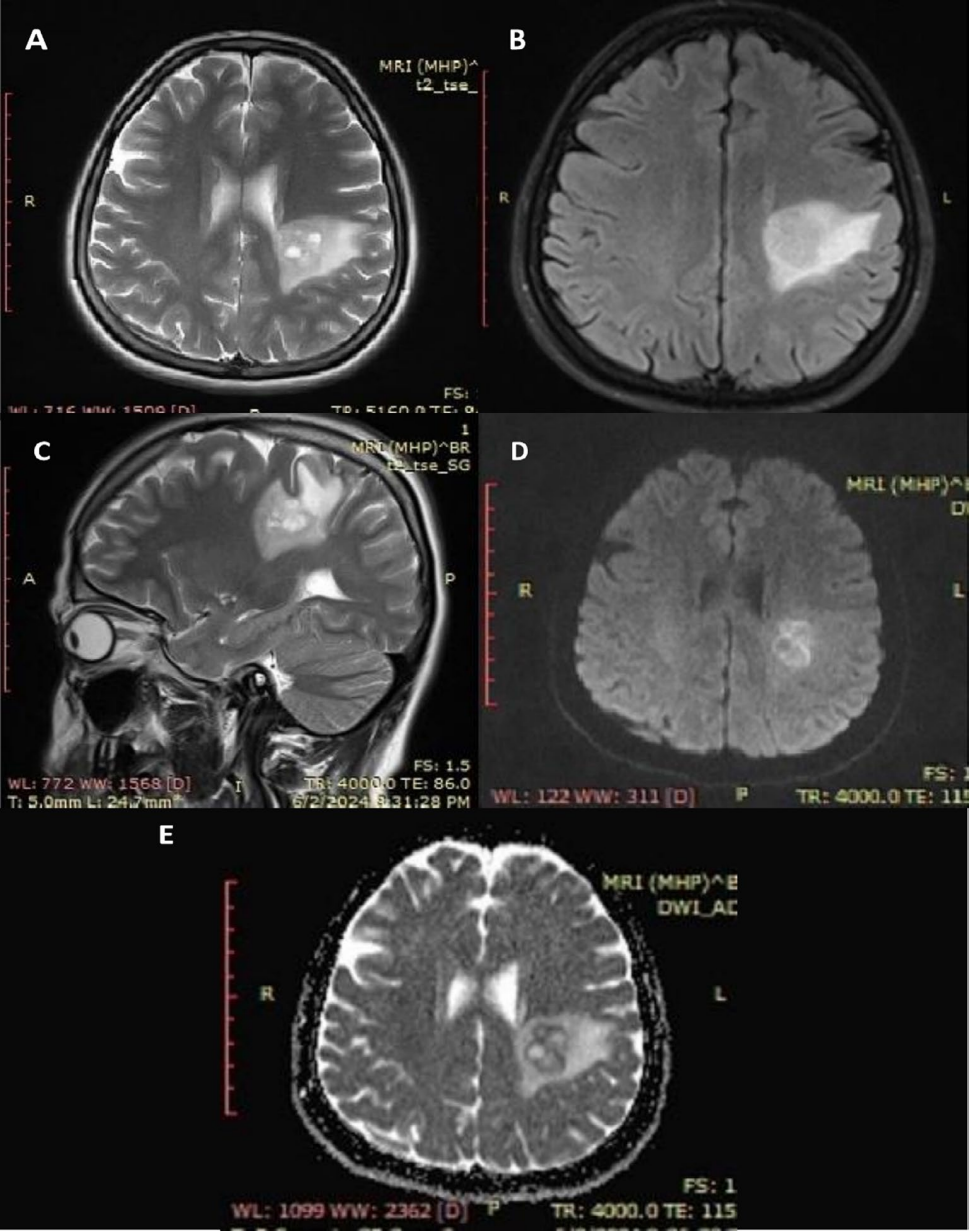
First brain MRI 2 months after initial presentation. (A) T2 Axial, (B) FLAIR Axial, (C) T2 Sagittal, (D) DWI, (E) ADC Map. T2 axial, sagittal and flair axial reveal left (LT) fronto‐parietal cystic mass surrounded by mild vasogenic edema. DWI and ADC images reveal diffusion restriction of the wall and the septa of the cystic mass.

**FIGURE 2:**
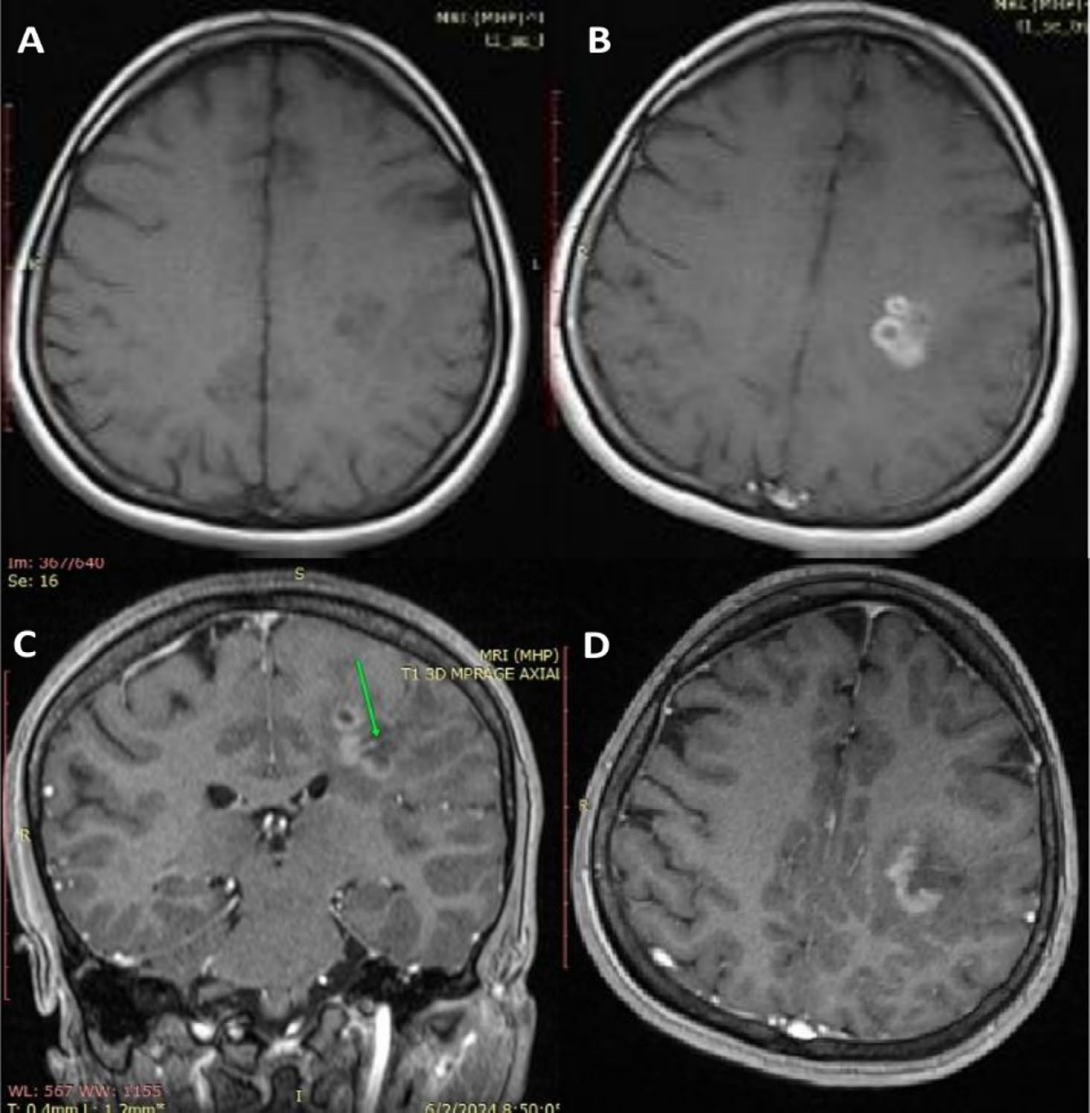
Contrast enhanced sequences of first brain MRI 2 months after initial presentation. (A) T1 Axial Pre Contrast, (B) T1 Axial Post Contrast, (C) T1 Coronal Post Contrast, (D) T1 Axial Post Contrast. At post contrast scan, both closed ring and open ring type of enhancement seen with open side of the ring facing gray matter, green arrow indicating there is an enhancing prominent medullary vein.

**FIGURE 3:**
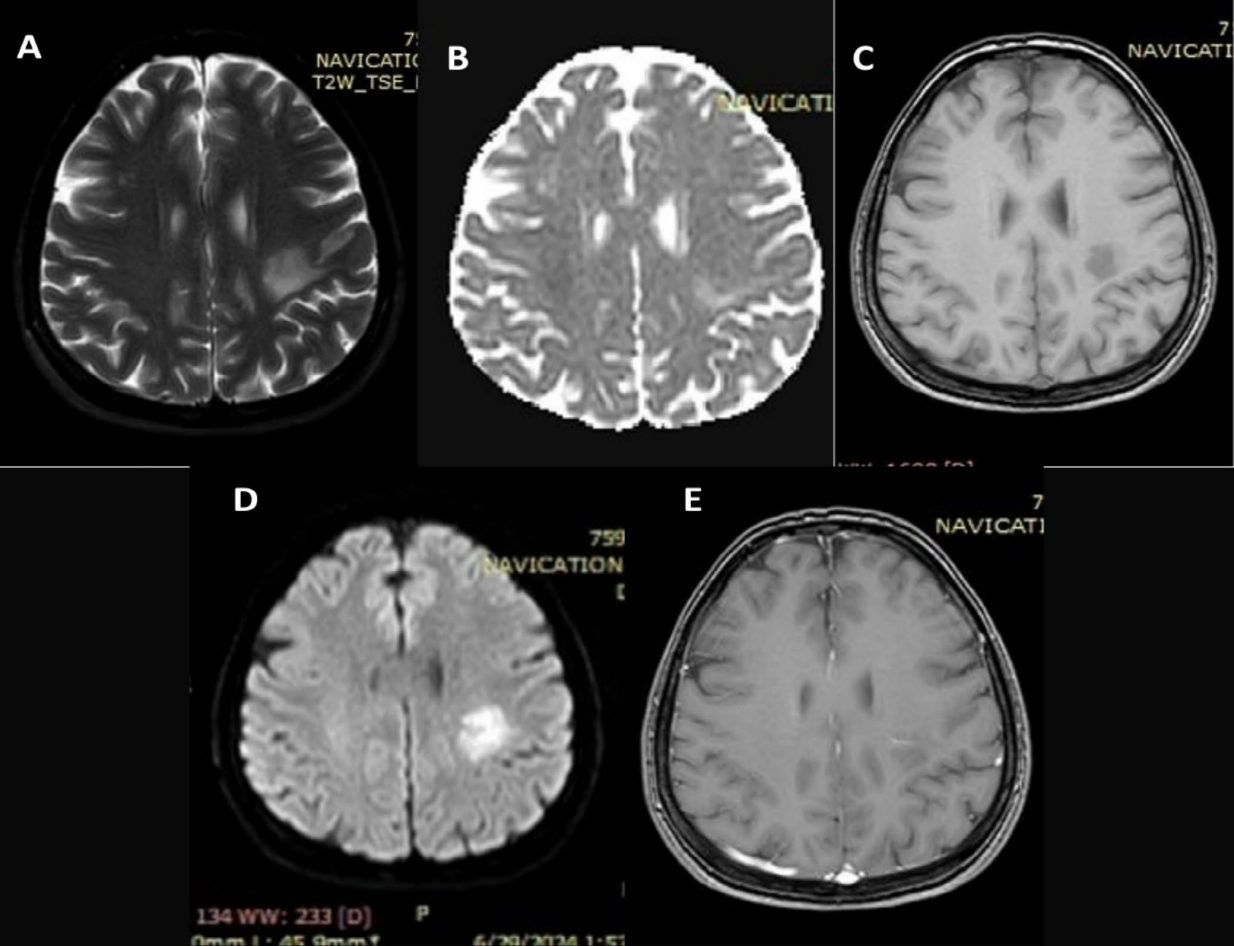
Second brain MRI 4 weeks after infliximab discontinuation and corticosteroid initiation. (A) T2 Axial, (B) ADC, (C) Flair Axial, (D) DWI, (E) T1 Axial Post Contrast. In this magnetic resonance imaging (MRI), regression of the previously enhancing cystic lesion is seen, leaving a small area of T2/FLAIR high SI 20 × 20 mm in size, no diffusion restriction seen nor any enhancement seen apart from the enhanced prominent medullary vein.

**FIGURE 4:**
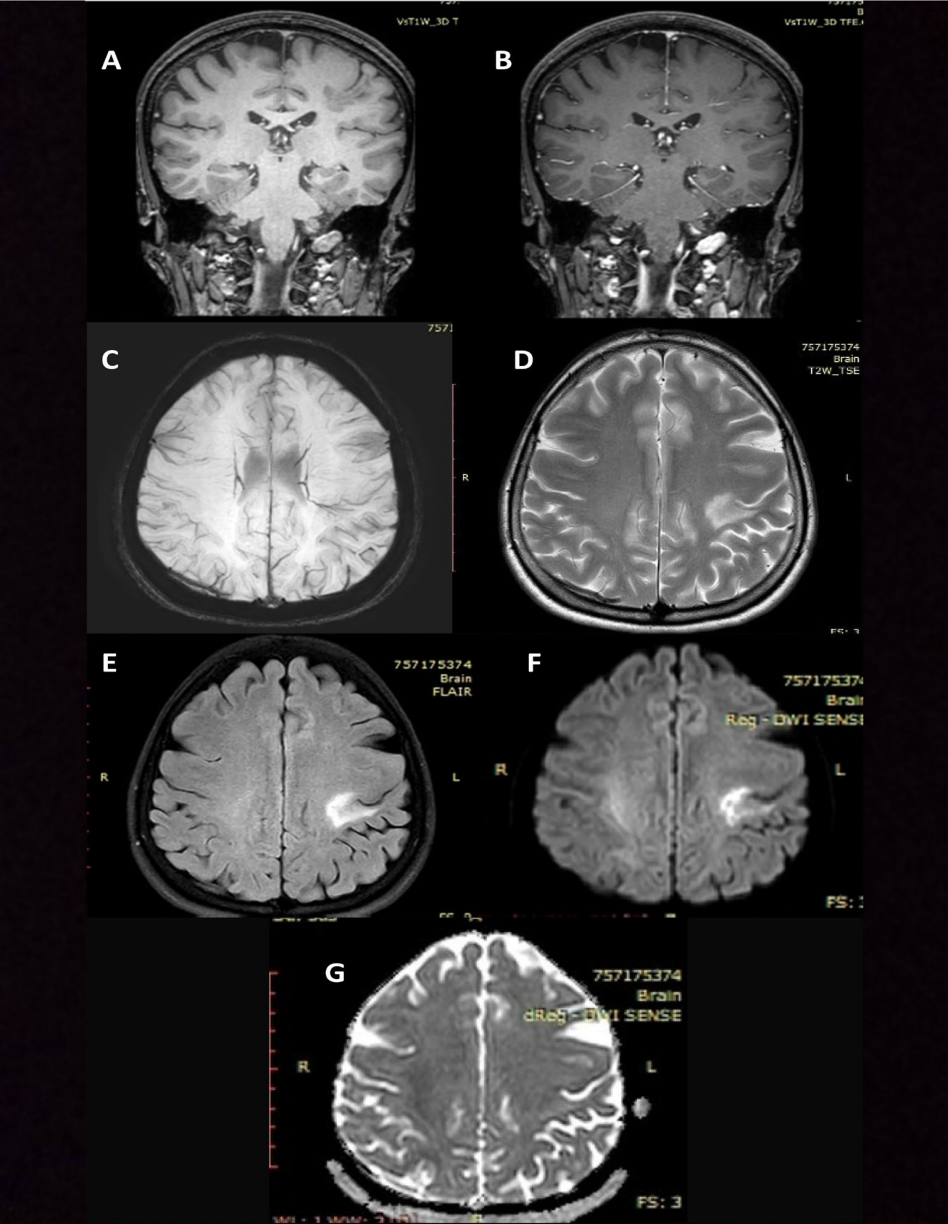
Third Brain MRI done after 5 months from first presentation. (A) T1 Coronal Pre Contrast, (B) T1 Coronal Post Contrast, (C) SWI, (D) T2 Axial, (E) FLAIR Axial, (F) DWI, (G) ADC. This magnetic resonance imaging (MRI) shows a residual area of T2w/flair white matter high signal intensity area of 16 × 15 mm in size, no enhancement and no diffusion restriction were seen, with prominent central vein sign as seen on susceptibility weighted imaging (SWI).

The first brain MRI was done 2 months after the initial presentation of symptoms as no baseline MRI was performed prior to the initiation of infliximab therapy. It revealed an LT frontoparietal periventricular white matter region lobulated outline cystic lesion measuring 22 × 16 × 16 mm in size (Figure 1). At the post‐contrast scan, both closed ring and open ring types of enhancement were seen, with the open side of the ring facing gray matter; there is an enhancing prominent medullary vein (Figure 2). After 4 weeks of discontinuation of infliximab and administration of methylprednisolone, a follow‐up MRI was conducted and revealed regression of the previously enhancing cystic lesion that left a small area of T2/FLAIR high SI (20 × 20 mm in size); no diffusion restriction nor any enhancement was seen apart from the enhanced prominent medullary vein (Figure 3). Three months after the first MRI, a follow‐up scan was conducted and showed a residual area of T2w/FLAIR white matter high signal intensity area (16 × 15 mm in size); no enhancement/diffusion restriction was seen, with prominent central vein sign seen on susceptibility‐weighted imaging (SWI) (Figure 4).

Infliximab was promptly discontinued after the MRI findings raised the possibility of infliximab‐induced CNS demyelination. The patient was treated with intravenous methylprednisolone (1.0 g/day for 5 days), which led to a mild improvement in her lower limb weakness and gait. Early discontinuation of the TNF antagonist and initiation of corticosteroids have been shown to improve outcomes in similar cases. All imaging features suggest TDL (Tumefactive demyelinating lesion) induced by infliximab with partial regression after discontinuation of infliximab and response to methylprednisolone. Methotrexate and hydroxychloroquine were reintroduced as immunosuppressive agents for the management of her rheumatoid arthritis. At the one‐year follow‐up, the patient demonstrated a favorable clinical response, with significant improvement in neurological function. However, she continued to report mild, persistent headaches.

In conclusion, the current report highlights the importance of careful observation of neurological symptoms and evaluation by MRI in patients receiving TNF‐α inhibitor therapy. While these agents have revolutionized RA management, they may pose risks to patients with underlying susceptibility to demyelinating diseases. Clinicians must maintain a high index of suspicion when patients present with new neurological symptoms during TNF‐α therapy and consider early discontinuation of the drug when demyelination is suspected. Patients with a positive family history of demyelination should undergo a neurological assessment before anti‐TNF treatment initiation. Ongoing research is necessary to understand the mechanisms underlying TNF‐α‐induced demyelination better and to identify strategies for minimizing this risk while preserving the therapeutic benefits of TNF antagonists. We suggest all patients should be clinically evaluated, including neurological assessment, before initiation of TNF‐α antagonist therapy to reduce the risk of treatment‐induced demyelinating events.

## 5 Discussion

TNF‐α is a proinflammatory cytokine regulating immune responses, inflammation, and tissue homeostasis [6, 7]. Consequently, this case adds to the growing evidence suggesting a link between TNF antagonists and CNS demyelination. While TNF‐α inhibitors have been highly effective in treating autoimmune diseases like RA, their effects on the CNS have raised concerns. Several mechanisms have been proposed to explain how TNF‐α inhibition could lead to demyelination, including:

1. Lack of Entry Theory: TNF‐α blockers cannot cross the intact blood–brain barrier (BBB) to prevent demyelination directly, yet they may increase demyelination by facilitating the entry of peripheral autoreactive T cells into the CNS [8].
2. TNFR2 Downregulation: TNF‐α also interacts with TNF receptor 2 (TNFR2), crucial in promoting the proliferation and differentiation of oligodendrocyte precursor cells involved in myelin repair. TNF‐α inhibitors may impair TNFR2 signaling, thereby hindering remyelination and exacerbating demyelination in patients receiving these treatments [9, 10].
3. Sponge Effect Theory: TNF‐α inhibitors block TNF‐α activity systemically but fail to suppress its activity within the CNS due to the impermeability of the BBB. As a result, TNF‐α may accumulate in the CNS, exacerbating neuroinflammation and promoting demyelination [8, 10].
4. Latent Infection Hypothesis: TNF‐α inhibitors have been known to unmask latent infections, which may trigger autoimmune processes leading to demyelination. This theory suggests that by suppressing the immune system, TNF antagonists may allow previously dormant pathogens or autoimmune reactions to emerge, leading to CNS damage [8, 10].

While demyelination is rare in patients receiving TNF‐α inhibitors, such cases highlight the importance of vigilance. The incidence of demyelinating disease in patients treated with TNF‐α inhibitors remains unknown [6]. Population‐based cohort studies from Denmark and Sweden revealed a lack of association between TNF‐α inhibitor treatment of RA and the risk of a neuroinflammatory event for the Swedish cohort and an elevated but not significantly increased risk in the Danish cohort [6, 11]. A non‐significant (*p* ≥ 0.05) increased risk of demyelinating events following exposure to TNF‐α inhibitors was reported in United States patients with RA in 2010 [6]. In general, demyelination is estimated to occur between 0.03% and 0.2% of patients receiving TNF‐α inhibitors [6, 12]. Tanno et al., in a case report, identified a potential link between TNFα inhibitor therapy, specifically infliximab, and the onset of CNS demyelination in patients with RA [13]. They presented two patients with refractory RA, previously treated with conventional therapies such as methotrexate and prednisone, who were started on infliximab. Despite initial improvements in RA symptoms and inflammatory markers, both patients developed new‐onset neurological symptoms indicative of CNS demyelination after multiple infliximab infusions. Notably, they found that steroid therapy effectively reduced both neurological symptoms and enhanced demyelinating lesions seen on MRI, suggesting a possible therapeutic approach in such cases [13].

In our case, the presence of a complex cystic brain lesion following infliximab therapy suggests that TNF‐α inhibitors may have played a role in triggering or exacerbating CNS demyelination, given that the patient didn't have a history of Multiple Sclerosis (MS). Discontinuation of infliximab and initiation of corticosteroid therapy led to clinical improvement, underscoring the importance of early detection and intervention in suspected TNF‐α‐induced neurological complications. The first MRI showed a LT frontoparietal multiloculated cystic lesion showing peripheral (wall) T2 wt high signal intensity with diffusion restriction, as well as both open and closed ring of enhancement seen at post‐contrast sequences with central vein sign, no mass effect seen but mild vasogenic oedema seen. After discontinuation of infliximab and administration of methylprednisolone, two MRIs were performed, revealing resolution of the previously seen cystic lesion with the disappearance of the diffusion restriction, enhancement, and surrounding oedema, just residual flair T2 high signal intensity was seen with a prominent central vein. In this regard, Ongphichetmetha et al. observed similar pre and post‐treatment MRI findings in patients with TDLs [14].

## Author Contributions

**Shahla Mohammed Saeed Rasul:** conceptualization, data curation, methodology, supervision, writing – review and editing. **Zana Abdulrahman Mohammed:** methodology, writing – original draft. **Aran Asaad Adiban:** methodology, writing – original draft. **Yahya Jawad Kadhm:** methodology, writing – original draft.

## Ethics Statement

The Ethics Committee of the College of Medicine, University of Sulaimani, Sulaimaniyah, Iraq, and the Institutional Review Board from the Shar Teaching Hospital, Sulaimaniyah, Iraq, approved a study protocol (No. 11/21 on May 15, 2023).

## Consent

Written informed consent was obtained from the patient before starting the study.

## Conflicts of Interest

The authors declare no conflicts of interest.

## Data Availability

The raw data of this study is available with the corresponding author and can be provided upon request.
